# 

**Published:** 1895-09

**Authors:** 


					﻿TME>
Southern Medical Record.
A Monthly Journal of Medicine and Surgery.
Vol. XXV. ATLANTA, GA.. SEPTEMBER, 1895.	No. 9.
BERNARD WOLFF, M. D., Editor and Proprietor.
Associate Editors :
A. W. GRIGGS, M. D.	WILLIS F. WESTMORELAND, M, D.
j. McFadden gaston, m. d.	louis h. jones, m. d.
Entered at the Post-office at Atlanta, Ga., as Second-Class Matter*
The Foote & Davies Co*, Atlanta, Ga.
				

